# Oral Administration of *Lactococcus lactis* Producing Interferon Type II, Enhances the Immune Response Against Bacterial Pathogens in Rainbow Trout

**DOI:** 10.3389/fimmu.2021.696803

**Published:** 2021-06-25

**Authors:** Alvaro Santibañez, Diego Paine, Mick Parra, Carlos Muñoz, Natalia Valdes, Claudia Zapata, Rodrigo Vargas, Alex Gonzalez, Mario Tello

**Affiliations:** ^1^ Departamento de Biología, Laboratorio de Metagenómica Bacteriana, Facultad de Química y Biología, Universidad de Santiago de Chile, Santiago, Chile; ^2^ Consorcio Tecnológico de Sanidad Acuícola, Ictio Biotechnologies S.A., Santiago, Chile; ^3^ Laboratorio de Microbiología Ambiental y Extremófilos, Departamento de Ciencias Biológicas, Universidad de los Lagos, Osorno, Chile; ^4^ IctioBiotic SpA, Santiago, Chile

**Keywords:** *Lactococcus lactis*, interferon type II, *Flavobacterium psychrophilum*, salmonids, oral administration

## Abstract

Lactic acid bacteria are a powerful vehicle for releasing of cytokines and immunostimulant peptides at the gastrointestinal level after oral administration. However, its therapeutic application against pathogens that affect rainbow trout and Atlantic salmon has been little explored. Type II interferon in Atlantic salmon activates the antiviral response, protecting against viral infection, but its role against bacterial infection has not been tested *in vivo.* In this work, through the design of a recombinant lactic acid bacterium capable of producing Interferon gamma from Atlantic salmon, we explore its role against bacterial infection and the ability to stimulate systemic immune response after oral administration of the recombinant probiotic. Recombinant interferon was active *in vitro*, mainly stimulating IL-6 expression in SHK-1 cells. *In vivo*, oral administration of the recombinant probiotic produced an increase in IL-6, IFNγ and IL-12 in the spleen and kidney, in addition to stimulating the activity of lysozyme in serum. The challenge trials indicated that the administration of the IFNγ-producing probiotic doubled the survival in fish infected with *F. psychrophilum*. In conclusion, our results showed that the oral administration of lactic acid bacteria producing IFNγ managed to stimulate the immune response at a systemic level, conferring protection against pathogens, showing a biotechnological potential for its application in aquaculture.

## Introduction

Interferons are a group of cytokines originally discovered by its antiviral properties (1). In mammals, these cytokines can be classified in three families according their homology, receptors, structure and function (2). Interferon types I and III are multi-gene families (17 type I and four type III) strongly induced by viruses, playing a major role controlling viral infection (3–5). Interferon type I also show a strong pro-apoptotic and anti-proliferative activity, being currently applied in anticancer and antiviral therapies (6).

In contrast, interferon type II or gamma Interferon (IFNγ) is a single gene encoded immunoregulatory cytokine, produced mainly by activated Natural Killer (NK) and T lymphocytes (T cells), although also can be secreted by B lymphocytes (B cells) and professional antigen-presenting cells (APCs) (7–9). Besides its antiviral properties, IFNγ also allows the activation of macrophages, improves the antigen presentation and induction of Major Histocompatibility Complex (MHC)-peptides complexes, regulates the T cell polarization, regulates the CD8 T cell homeostasis, stimulates apoptosis and improves the antimicrobial mechanism against intracellular bacteria or parasites (8, 10–12). IFNγ is clinically used against infection in patients with Chronic Granulomatous Disease, although it is also effective against *Mycobacterium tuberculosis*, Salmonella and fungal infection (13–16).

Teleost and mammalian immune systems show several parallels in innate and acquired immunity. Bioactivity assays using recombinant IFNγ from *Salmo salar* or *Oncorhynchus mykiss* have shown this cytokine is able to induce the expression of MHC-I, MHC-II and proteins related to the processing of the antigens and increase the respiratory burst of phagocytic cells (17–20). *In vitro*, IFNγ from *S. salar* also has antiviral properties against Salmonid Alphavirus (SAV) and Infectious Pancreatic Necrosis Virus (IPNV) (21). Moreover, in *O. mykiss* cells, IFNγ is able to reduce *in vitro* the load of *Piscirickettsia salmonis*, suggesting that in salmonids, as in mammals, IFNγ also has antibacterial properties (22).

IFNγ has the potential to be used as a therapeutic or prophylactic treatment against bacterial and viral pathogens from salmonid aquaculture. However, the application of these cytokines has been limited due to the lack of a feasible vehicle of delivery compatible with the fish physiology.

In the last decades, Lactic Acid Bacteria (LAB) have risen as a feasible vehicle for the *in situ* delivery of cytokines and bioactive peptides inside of the gastrointestinal tract of mammalians (23, 24). Alpha Interferon (IFNα) (25–27), beta Interferon (IFNβ) (28), IFNγ (29, 30), and IL-10 (31) have been successfully expressed in LAB under its biologically functional form, producing local and systemic effects after administration to animals.

In *S. salar* and *O. mykiss* the use of LAB for *in situ* delivery of bioactive proteins has been poorly explored, being used only for delivery of antigenic peptides (32, 33). In this work we use *Lactococcus lactis* for the expression of a functional IFNγ from *S. salar*. The oral administration of this modified LAB reduces the susceptibility of treated fish against *F. psychrophilum* infection, an important bacterium that affects the salmonid industry.

## Materials and Methods

### Construction of MT009 Strain

The gene encoding for the recombinant IFNγ from *S. salar* (rIFNγ) was designed *in silico* using the protein sequence of type II interferon from *S. salar* (FJ263446.1), the Usp45 Signal peptide (SP), the tag GGGHHHHHH, and the promotor P1 of *L. lactis*. The Usp45 SP, type II interferon and tag sequence, were arranged in frame downstream of the P1 promoter. The sequence was synthesized in GenScript and codogenically optimized according to the codon usage of *L. lactis*. This sequence was cloned in the pNZ8149 plasmid (MoBiTec GmbH, Germany) between the sites NcoI and XbaI. The ligation product was electroporated into *L. lactis* NZ3900. The identity of the new construct, prIFNγ, was corroborated by sequencing. The *L. lactis* strain NZ3900 containing the plasmid prIFNγ, was named MT009.

The preparation of electrocompetent *L. lactis* NZ3900 cells was performed based on the protocol suggested by Mobitec GmbH (34). A colony of *L. lactis* NZ3900 was inoculated in 5 ml of SG-GM17 medium (M17 medium containing 0.5 M sucrose, 2.5% glycine, and 0.5% glucose) and cultured overnight at 30°C without agitation. The culture was inoculated in 40 ml of SG-GM17 medium and grown for 16 h at 30°C without shaking. The next day, this culture was inoculated in 400 ml of SG-GM17 medium and grown to an OD600 between 0.2 and 0.3. Subsequently, the culture was centrifuged at 6,000×*g* for 20 min at 4°C and the collected pellet was washed three times in cold wash buffer (0.5 M sucrose, 10% glycerol, 4°C). In each step, the pellet was collected by centrifugation at 6,000×*g* for 20 min at 4°C and was resuspended by vortex in the corresponding buffer. After the final wash, the pellet was resuspended in 3 ml of wash buffer, aliquoted into 200 µl fractions, and then stored at −80°C.

### MT009 Culture Conditions for Hybridization and Biological Activity Assays

From an isolated colony of MT009 a pre-culture was prepared in 0.5% M17-Lactose medium, after incubating overnight, this culture was used to inoculate (2%) 40 ml of M17-Lactose 0.5% medium. After reaching an optical density at 600 nm between 0.4 and 0.6, the culture was induced with nisin 10 ng/ml for 2 h. Bacteria were separated from the supernatant by centrifugation at 6,000×*g* for 20 min at 4°C. The obtained pellet was used in western blot and biological functionality tests, while the supernatant was used in dot blot assays. The MT009 cultures were carried out at 30°C without shaking.

### Preparation of Cytoplasmic Extracts

From the MT009 culture, the bacterial pellet was resuspended in 1 ml of 1× PBS supplemented with 1 mM protease inhibitor Phenylmethylsulfonyl fluoride (PMSF). For their rupture, the resuspended cells were kept on ice and sonicated (ultrasonic processor, Sonic Vibracell) for 2 min divided into EIGHT pulses (130 W, 20 kHz, 100%, 2 mm Cv188 stem) of 15 s with intervals of 1 min. After treatment, the cellular debris was separated by centrifugation (13,000×*g*, for 10 min at 4°C). The supernatant containing the cytoplasmic proteins was removed and stored at −20°C. Total protein concentration was determined by the Bradford method.

### SDS-PAGE and Western Blot

For the detection of rIFNγ in cytoplasmic extracts, proteins were separated according to their mass by SDS-PAGE (Stacking gel: 8% Acrylamide/Bisacrylamide 29: 1, pH 6.8; Resolving gel: 10% Acrylamide/Bisacrylamide 29: 1, pH 8.8). Ten µg of total protein extracts from MT009 and control strains MT005 (*L. lactis* NZ3900 with pNZ8149) were loaded, together with the BenchMark ™ His-tagged Protein Standard. The samples were subjected to electrophoresis for 90 min at 100 V. After electrophoresis the proteins were electrotransferred (300 mA for 2 h at 16°C) to a nitrocellulose membrane. Subsequently, the membrane was blocked for 1 h in a 2% BSA solution and washed three times with 1× PBS-Tween 20 (0.5%). The membrane was incubated with Rabbit polyclonal anti-His antibody (Abcam) (dilution 1/5,000) for 1 h at 37°C and then washed three times with PBS-Tween 20 (0.5%). Subsequently, the membrane was incubated with the polyclonal anti-rabbit IgG antibody conjugated to Horseradish Peroxidase (HRP) (1/5,000 dilution) for 1 h at 37°C. Then the membrane was washed three times with PBS-Tween 20 (0.5%). The membrane was incubated with 10 ml chemiluminescent developer solution (Pierce™ ECL Western Blotting Substrate) and exposed to photographic film. For the detection of rIFNγ in the extracellular protein concentrate, a dot blot was performed. The nitrocellulose membrane was loaded with 10 µl of extracts to give 1 µg of total protein per sample. Once air-dried, the membrane was hybridized and developed following the same protocol described for the western blot.

### Culture Cells

Cultures of the Atlantic salmon head kidney cell line (SHK-1) (Sigma-Aldrich) were prepared up to 80% confluence in L15 medium supplemented with 4 mM glutamine and 5% fetal bovine serum. These cultures were incubated for 8 h at 16°C with 0 to 200 ng/ml of *L. lactis* cytoplasmic extracts containing rIFNγ (MT009). The total protein concentration was adjusted to the same value with cytoplasmic protein extract of the control strain MT005. After the treatment with cytoplasmic extracts, SHK-1 cell cultures were processed to extract total RNA.

### RT-qPCR

Total RNA from cell cultures was extracted using total RNA kit (Omega Bio-tek), while the total RNA from spleen and kidney was extracted with TRIZOL using approximately 30 mg of each organ. In both cases total RNA integrity was evaluated using agarose gels (1%, TAE 1X) and quantified by absorbance at 260 nm, using the Tecan Infinite 200 PRO equipment or a Synergy™ 2.0 multi-well reader (Biotek). The RNA (2 μg) was treated with 1 U of RQ1 RNase-Free DNase (Promega, USA) for 30 min at 37°C in a 10 μl volume. The RNase-Free DNase was inactivated adding 1 μl of 25 mM EDTA and incubating at 65°C during 10 min. The RT reaction was performed with 1 µg of total RNA previously treated with RNase-Free DNase, 200 Units of M-MLV reverse transcriptase (Promega, USA) and 100 pmol of oligo-dT 18-mer, in a 25 μl volume. The RT reaction was performed at 42°C for 1 h and stopped by denaturation at 70°C for 15 min. The qPCR reaction was set up using the SYBR Fast Universal qPCR kit (Kapa Biosystem USA), in a 20 μl volume, using 2 μl of the RT reaction, and 10 pmol of each primer. To detect and quantify expression of Stat-1, gamma IP10, IFNγ, IL-1β, TNFα, TGFβ, IL-12, IL-6, Mx and eF1α, the thermal program consisted of 40 cycles of 15 s at 95°C, 15 s at the annealing temperature, and 30 s at 72°C. The qPCR reactions were performed in duplicate on a Stratagene Mx3000P qPCR cycler. The expression of the genes was normalized with respect to the control condition and the expression of the eF1α gene using the ΔΔCt method described by Pfaffl (35). The primers used together the annealing temperature are listed in **Table 1**.

**Table 1:** Primers used in qPCR experiments.

Gene	Sequence	Ta	Ref
EF1a	F: 5’-GGGTGAGTTTGAGGCTGGTA-3’ R: 5’-TTCTGGATCTCCTCAAACCG-3’	60°C	(36)
IL-12	F: 5’-TGACGCTTTTTCTCACCGGTTGT-3’ R: 5’-ACGCTTTGCAGCATGAGCTTGA-3’	60°C	(37)
IFN-γ	F: 5’-CCGTACACCGATTGAGGACT-3’ R: 5’-GCGGCATTACTCCATCCTAA-3’	60°C	(36)
TGF-β	F: 5’-AGCTCTCGGAAGAAACGACA-3’ R: 5’-AGTAGCCAGTGGGTTCATGG-3’	60°C	(36)
IL-1β	F: 5’-CCCCATTGAGACTAAAGCCA-3’ R: 5’-GCAACCTCCTCTAGGTGCAG-3’	60°C	(36)
IL-6	F: 5’- CCTTGCGGAACCAACAGTTTG-3’ R: 5’- CCTCAGCAACCTTCATCTGGTC- 3’	60°C	(38)
STAT-1	F: 5’- GACCAGCGAACCCAAGAACCTGAA-3’ R: 5’-CACAAAGCCCAGGATGCAACCAT-3’	60°C	(38)
Gamma IP-10	F: 5’-GTGTCTGAATCCAGAGGCTCCA-3’ R: 5’-TCTCATGGTGCTCTCTGTTCCA-3’	60°C	This work
rpoC	F: 5’-AGG GAG ACT GCC GGT GAT A-3’ R: 5’-ACTACGAGGCGCTTTCTCA-3’	55°C	(39)

### Oral Administration of MT009 and Challenge Trials

To analyze the expression of genes that respond to IFNγ during oral administration of MT009, *O. mykiss* of approximately 25 g were placed in two experimental groups of 12 fish into 27 l tanks. After 7 d of acclimatation, one group was fed with food supplemented with MT009 and the other group continued with a normal diet. Samples of three fish were taken from each group at the beginning of the experiment (T0) and at 2, 4 and 6 d during the treatment with MT009. Each sampled fish was euthanized (see section *Fish Maintenance and Euthanize Protocols*) and processed to extract blood, spleen and kidney.

To analyze the effect of MT009 on the expression of genes that respond to IFNγ after oral administration, we followed a similar design as above, with fish placed into three experimental groups of 12 fish into 27 l tanks. After one week of acclimatation, one group was fed 7 d with feed supplemented with MT009, the second group was fed with feed supplemented with the control strain (MT005) and the third group continued with a normal diet. After administration of MT005 and MT009 fish were fed with normal feed. Samples of three fish were taken from each group at 1, 3, 5 and 7 d after the end of the treatment with the test strain. The fish were euthanized and processed to extract blood, spleen and kidney tissues.

For the challenge assay with *Flavobacterium psychrophilum* strain 10094 (ETECMA) (40), six tanks of 27 l with 12 specimens of *O. mykiss* of approximately 25 g were arranged in three experimental groups as for the feed trials above. The 6th day post treatment, the groups fed with MT005 (one tank) and MT009 (two tanks), and the untreated fish (two tanks) were challenges by intraperitoneal injection with 10^8^ CFU of *F. psychrophilum* suspended in 100 μl of sterile PBS. As control, one tank of untreated fish was injected with 100 μl of sterile PBS.

The RPS of the MT009 treatment was calculated according to the following formula:

$$\text{RPS}=\left(1-\;\frac{{\text{\%M}}^{\text{MT}009}}{{\text{\%M}}^{\text{NF}}}\right)\times \;100$$

Where %M^MT009^ is the percentage of mortality of the group fed with MT009 and %M^NF^ is the percentage of mortality of the group fed with normal feed.

The *F. psychrophilum* load in surviving fish was determined by qPCR using absolute quantification of *rpoC* gene with a standard curve. Approximately 30 mg of spleen were processed to extract total DNA using Wizard^®^ Genomic DNA Purification Kit (Promega). The DNA was quantified with an Infinite^®^ 200 PRO NanoQuant (TECAN) and adjusted to 50 ng/μl. The qPCR was set up using the SensiFAST™ SYBR^®^ No-ROX Kit (Bioline), in a 10 μl volume, using 50 ng of DNA from spleen, 2.5 pmol of rpoC-Fp forward primer, and 2.5 pmol of rpoC-Fp reverse primer (**Table 1**) (39). The thermal program consisted of one initial denaturation of 3 min at 95°C followed by 35 cycles of 30 s at 95°C, 15 s at 55°C, and 20 s at 72°C. The standard curve was set up using a plasmid that contain cloned the *rpoC* gene of *F. psychrophilum* (the plasmid was donated by Ictio Biotechnologies S.A). The plasmid was diluted in a range between 10^1^ and 10^9^ copies/μl.

### Lysozyme Activity

The lysozyme activity in serum was evaluated using the *Micrococcus lysodeikticus* assay. Samples of blood were coagulated at room temperature and centrifugated at 6,000×*g* for 15 min at 4°C. The serum was separated in a clean tube and stored at −80°C. Lysozyme activity was measured according to the protocol described by Shugar (41) adapted in a microtiter plate. Briefly, 20 μl of serum was mixed with 180 μl of 0.015% [w/v] *M. lysodeikticus* cell suspension in 50 mM Potassium Phosphate Buffer, pH 6.24. The changes in the A_450_ of samples (S) and blank (B) were measured for 5 min. The slopes (Δ450/min) of sample and blank were used to calculate the lysozyme activity according to the following formula:

$$\text{Lysozyme activity}\;\left[\frac{\text{U}}{\text{ml}}\;\right]=\frac{\left(\frac{{\text{ΔA}}_{450}^{\text{S}}}{\text{min}}-\frac{{\text{ΔA}}_{450}^{\text{B}}}{\text{min}}\right)\times \text{Df}}{2\times 10^{-5\;}\left[\text{ml}\right]}$$

### Preparation of the Pathogen Inoculum for Challenge Assays

For the challenge assay with *F. psychrophilum* strain 10094, the bacteria were grown in 10 ml of Tryptone Yeast Extract Salts (TYES) medium pH 7.2 at 15°C with constant agitation (200 rpm) for 72 h. Five milliliters of this pre-inoculum were used to inoculate 95 ml of sterile TYES broth pH 7.2, then the bacteria were cultured for 4 d at 15°C with constant agitation (200 rpm). At the end of the culture final density was adjusted with TYES to 10^9^ CFU/ml and 100 μl of this culture was used to infect the fish intraperitoneally. *F. psychrophilum* strain 10094 was isolated on November 6th, 2015 from a specimen of *O. mykiss* in Chiloé, Chile.

### Fish Maintenance and Euthanize Protocols

Fish were acclimated for one week before treatment at 12°C in freshwater aquariums with a biomass not higher than 14 g/l, with continuous aeration, and fed with commercial pellets (EWOS MICRO™ 2 mm) at 1% of body weight. The fish were maintained in freshwater with a pH between 6.6 and 7, the salinity was adjusted to 6 PSU with NaCl to prevent fungal infection, and total ammonia was maintained in a range below 0.02 mg/l. Seventy percent of the water in all the aquaria were changed every day after feeding. Water parameters were monitored daily prior to and after changing the water. Feeding, changing the water, and measuring water parameters were all done manually. The *L. lactis* strains (MT009 or MT005) were administered to the fish together with feed. The bacterial pellet of the cultures was washed with 1 ml of M17 medium, collected by centrifugation at 6,000×*g* for 10 min at 4° C and resuspended in M17 medium in a volume equivalent to 1/10 of the volume of the original culture. The bacteria suspension was mixed with edible oil in a 2:1 ratio and emulsified by vortexing. The emulsion obtained was mixed with the feed, homogenized by shaking in a plastic container and incubated for 1 h at 30°C to allow absorption and drying on feed pellets.

To avoid unnecessary suffering of fish during the challenge and sampling, fish were anesthetized with benzocaine 40 mg/l no longer than 2 min before the intraperitoneal injection, while fish sampled were euthanized with an overdose of anesthesia with benzocaine 40 mg/l for 5–10 min. The fish were maintained in accordance with the ethical standards of the Institutional Ethics Committee of the Universidad de Santiago de Chile and the relevant legislation in force.

## Results

### Expression of rIFNγ in *L. lactis* NZ3900

The interferon type II encoded in the *S. salar* genome is a 21.2 kDa protein in its immature form. The modification introduced to allow its expression and secretion in *L. lactis*, included the replacement of the eukaryotic signal peptide (SP) by the prokaryotic SP of Usp45 protein, the introduction of a GGGHHHHHH tag in the C-terminal, the codon optimization to the codon usage of *L. lactis* and the inclusion of a P1 promoter to allow a basal expression. This *in silico* design produces a theoretical protein (rIFNγ) of 22.2 kDa. This sequence was synthesized and cloned into pNZ8149 using *L. lactis* NZ3900 as host. The plasmid containing the rIFNγ gene in pNZ8149 was named prIFNγ and the strain of the *L. lactis* containing this plasmid was named MT009.

**Figure 1:**
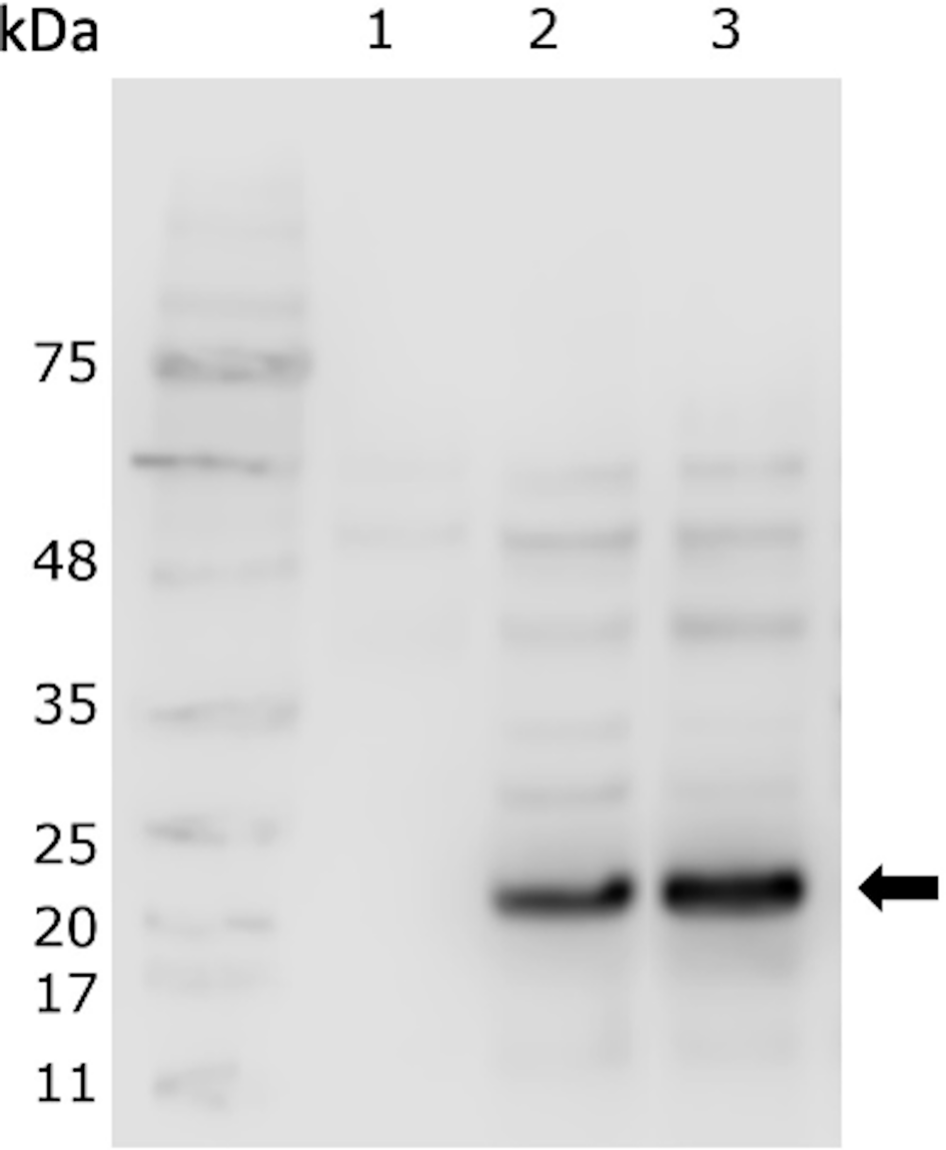
Presence of rIFNγ in the cytoplasmic extract of MT009. The figure shows a western blot using anti-histag and the cytoplasmic extract from cultures of MT005 (1), MT009 without induction (2) and induced with nisin 5 ng/ml (3). The arrow indicates the band of ∼23 kDa corresponding to the rIFNγ. The effects of the nisin from 1 ng/ml and 10 ng/ml are shown in the **Supplementary Figure 1**.

The expression of rIFNγ was evaluated in the cytoplasmic and in extracellular extracts of the MT009 strain. The expression was evaluated in the presence of different concentration of nisin (1, 5, and 10 ng/ml), which is the inducer of the pNIS promoter present in the plasmid pNZ8149. We also analyzed the expression of rIFNγ in cultures of MT009 without nisin. As a negative control, we used the cytoplasmic or extracellular extract prepared from cultures of the MT005 strain (*L. lactis* NZ3900). We identified a protein of approximately 23–24 kDa close to the theoretical 22.2 kDa. This protein was present in the cytoplasmic extract from MT009 with and without presence of the inducer, suggesting that promoter P1 acts constitutively and is able to quench the pNIS promoter (**Figure 1**). When the supernatant was analyzed, no band was identified, so the supernatant was concentrated and, after this, a thin band at 21 kDa was identified (data not shown), suggesting poor extracellular secretion.

### Bioactivity of rIFNγ

**Figure 2:**
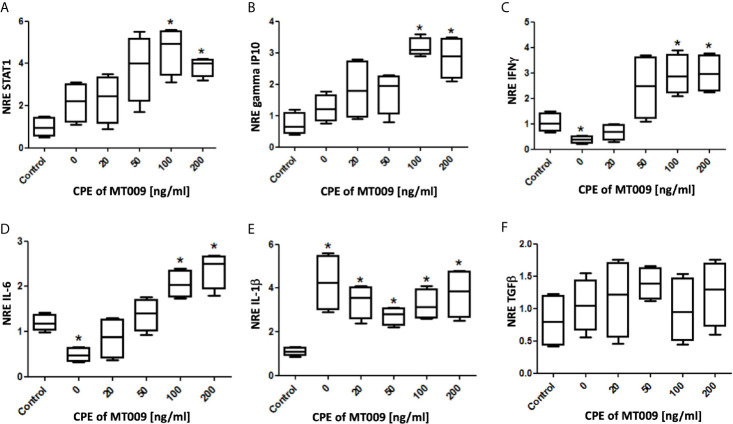
Effect of MT009 protein extract on the expression of IFNγ related genes in SHK-1 cells. The figure shows the relative expression of genes related with the downstream IFNγ-response pathway Stat-1 **(A)**, gamma IP10 **(B)**, IFNγ **(C)** and IL-6 **(D)** after exposure to the cytoplasmic extract of MT009 (*L. lactis* NZ3900 prIFNγ). The figure also shows the effects of these extracts on the expression of **(E)** inflammatory (IL-1β) and **(F)** anti-inflammatory (TGF-β) cytokines. The expression was normalized with respect to the expression of eF1α (NRE). The concentration of bacterial cytoplasmic proteins extracts (CPE) was adjusted with extracts of MT005 (*L. lactis* pNZ8149) to 200 ng/ml. The NRE of each gene was compared against the NRE of the control (cells without treatment). The assays were performed using biological and technical replicates. The significance was analyzed using the Mann–Whitney test (*p < 0.05).

In order to test if the rIFNγ present in the cytoplasmic extracts was functional, we designed a test using SHK-1 cell cultures. These cells were exposed to several concentrations (0 to 200 ng/ml) of cytoplasmic protein extracted from MT009 cultures. To control the effect on the cells of different concentrations of bacterial proteins from cytoplasm of *L. lactis* NZ3900, the total concentration of cytoplasmic bacterial proteins was adjusted to 200 ng/ml using cytoplasmic protein extracts from MT005 cultures grown under the same condition. We evaluated the effect over the expression of genes under the activation pathway of IFNγ, STAT1, gamma IP10, IL-6 and IFNγ. We also evaluated the effect on the expression of general anti-inflammatory and inflammatory cytokines TGF-β and IL-1β, respectively. We observed a dose dependent tendency in the expression of all the genes that were downstream in the pathway of genes activated by IFNγ, particularly in the expression of IFNγ and IL-6. We also observed that the cytoplasmic extracts of MT009 showed no effects on the expression of anti-inflammatory cytokine TGF-β, and in the increase of the expression of IL-1β suggesting that cells respond to other bacterial components present in the MT009 protein extract (**Figure 2**).

### *In vivo* Effects During the Oral Administration of MT009 on the Expression of IFNγ Related Genes

**Figure 3:**
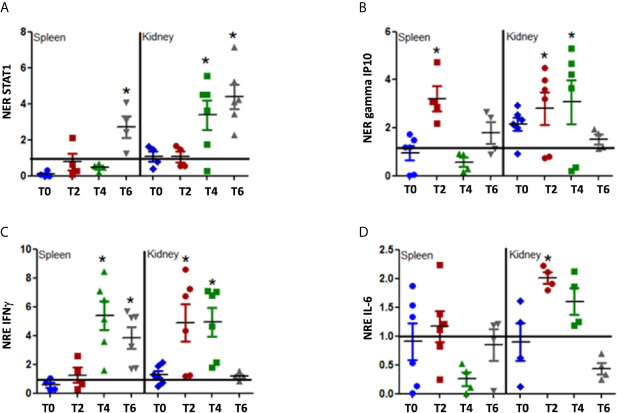
Expression of IFNγ related genes during feeding with MT009. The figure shows the relative expression of STAT1 **(A)**, gamma IP10 **(B)**, IFNγ **(C)** and IL-6 **(D)**. The expression was evaluated in the spleen and kidney at the beginning of the treatment (T0) and every two days during treatment (T2, T4, T6). The expression was normalized with respect to the expression of eF1α gene and by the expression of the genes in the control condition at the same time point. The expression values were compared with the expression at T0. The significance was analyzed using the Mann–Whitney test (*p < 0.05).

Our initial results showed that rIFNγ was biologically active *in vitro* stimulating the genes related with the IFNγ response. To determine if the MT009 strain stimulate the interferon γ pathway when administered orally to rainbow trout, we designed an experiment where fish were fed with 10^7^ CFU of MT009 per day for one week. Tissues from fish were taken every 2 d during the feeding. The expression of IFNγ, STAT-1, gamma IP-10, and IL-6 was evaluated in the spleen and kidney. We observed an increase in the expression of IFNγ in kidney between days 2 and 4 after MT009 feeding began, returning to the initial values on day 6. In the spleen, the increase in the expression of IFNγ began later, on day 4 and remained high until day 6. A similar pattern was observed with gamma IP10 which increased its expression in the kidney between days 2 and 4, and returned to values close to those observed in the control fish on day 6. In the spleen, we observed an increase in the expression of gamma IP10 only on day 2. STAT-1 also increased its expression mainly in kidney after day 4. IL-6 only increased its expression in kidney, such as is observed in SHK-1 cells (**Figure 3**). MT009 also increased the expression of IL-12, a cytokine secreted by macrophages in response to IFNγ, in the spleen and in a lower extent in kidney (**Supplementary Figure 2B**). Interestingly, the expression on IL-1β showed a slight increase in the spleen throughout the time analyzed, but in the kidney showed a strong increase after day 2 (**Supplementary Figure 2A**). Our results show that the kidney and spleen responded differentially to MT009, with the kidney an organ with a more robust and complex response than the spleen.

### *In Vivo* Effects Post Oral Administration of MT009 on the Expression of IFNγ Related Genes

**Figure 4:**
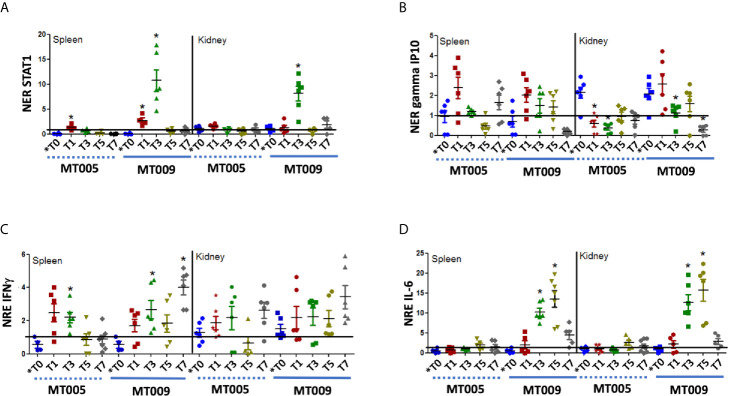
Expression of IFNγ related genes after oral administration of MT009 and MT005. The figure shows the relative expression of STAT1 **(A)**, gamma IP10 **(B)**, IFNγ **(C)**, and IL-6 **(D)** in fish treated with MT009 (solid blue line) and fish treated with the *L. lactis* strain containing the plasmid pNZ8149 (MT005) (dashed blue line). Gene expression was evaluated in the spleen and kidney at the beginning of the treatment (*T0) and every two days after treatment (T1, T3, T5, T7). The expression was normalized with respect to the expression of eF1α gene and by the expression of the genes in the control condition at the same time point. The expression values were compared with the expression at T0. The significance was analyzed using the Mann–Whitney test (*p < 0.05).

The previous results show that MT009 was able to induce the expression of genes related to the IFNγ pathway. These effects could be due to the bacterial host or the associated rIFNγ. In order to determine whether MT009 influenced the immune system due to prIFNγ and if these effects are present post feed with MT009, we evaluated the expression of IFNγ, STAT-1, IL-6 and gamma IP10 in the spleen and kidney of fish fed with feed supplemented with MT009, MT005 (*L. lactis* NZ3900 with pNZ8149) and without any bacterial supplementation. Samples were taken every 2 d for a week, starting the first day after feeding with MT005 or MT009. Our results showed that *L. lactis* was also able to induce the expression of IFNγ after feeding, but in the fish fed with *L. lactis* expressing rIFNγ, the induction of IFNγ remained high on day 7 post feeding compared to MT005 (∼4 fold in spleen and ∼1.2 fold in kidney). A similar pattern was observed with the expression of gamma IP10. A more prominent effect was observed in the expression of STAT-1 and IL-6 where maximum expression was observed on days 3 and 5 post treatment, respectively. This effect was observed in spleen and kidney and in both the expression decreased drastically on day 7 post treatment (**Figure 4**). Interestingly, both MT005 and MT009 were able to induce the expression of TGF-β, suggesting that the *L. lactis* possesses anti-inflammatory properties, besides the increase in the expression of IL-6 (**Supplementary Figure 3**).

### Effect of MT009 on the Lysozyme Activity in Serum After Treatment

**Figure 5:**
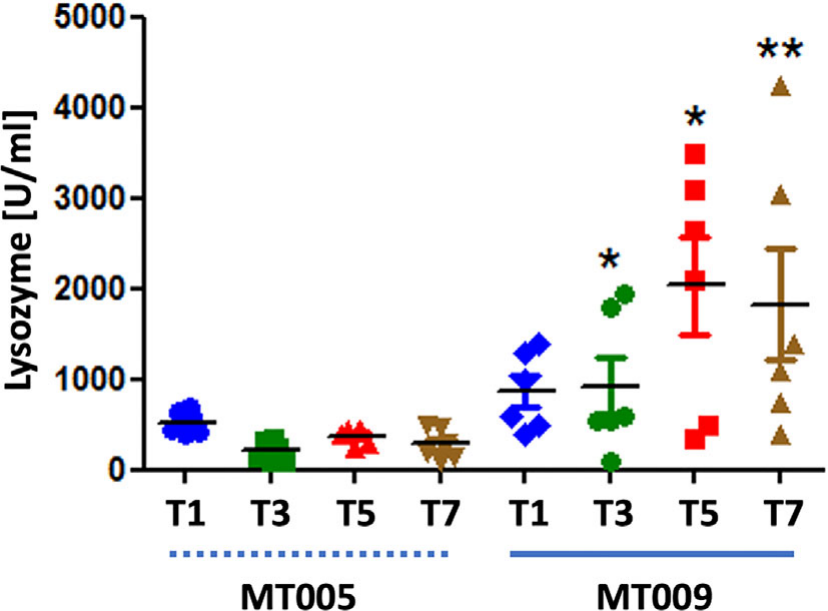
Lysozyme activity in serum post treatment. The figure shows the lysozyme activity in serum of fish treated during seven days with MT009 (solid blue line) and MT005 (dashed blue line). Samples were taken at 1, 3, 5 and 7-days post treatment (PT). The assay was performed using three fish per day and the lysozyme activity was measure in triplicated. The significance was analyzed using the Mann–Whitney test (*p < 0.05, **p < 0.01).

The previous finding indicated that IL-6 was strongly induced after treatment with MT009 and this effect was not present in the parental strain MT005, suggesting that is an effect related to the production of IFNγ by MT009. Taken into account that IFNγ and IL-6 induce the expression of lysozyme in mammals, we evaluated lysozyme activity in serum after treatment with MT009 and MT005. Our results showed a strong induction of the lysozyme activity in serum after treatment with MT009, but not with MT005, indicating that the induction of lysozyme activity is related to the presence of the prIFNγ in the strain MT009 (**Figure 5**). This induction was not observed in serum during the initial administration of MT009 (**Supplementary Figure 4**), suggesting that induction of lysozyme activity is consequence of a complex cascade and is probably achieved by the constant accumulation in serum due to the stimulation of the immune system.

### Protective Activity of MT009 Against Bacterial Infection

**Figure 6:**
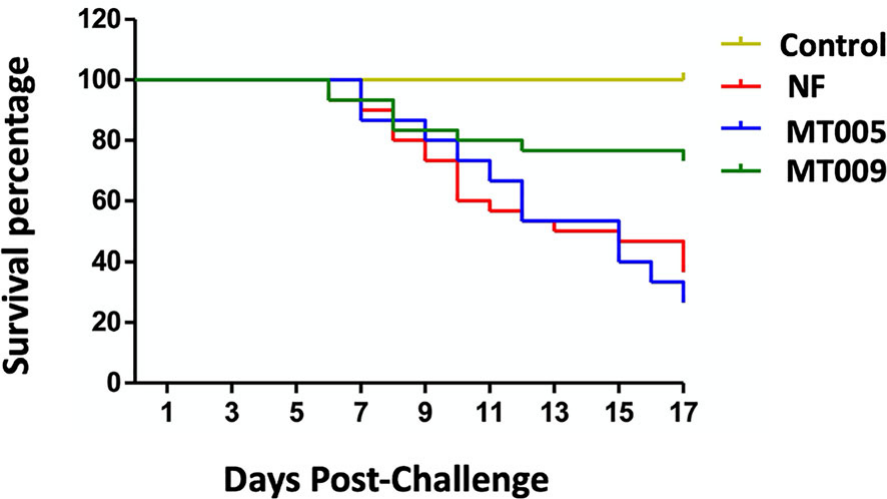
Challenge assay with *Flavobacterium psychrophilum* strain 10094. The figure shows the Kaplan–Meier survival plot of *Oncorhynchus mykiss* specimens challenged intraperitoneally with 10^8^ CFU of *F. psychrophilum* strain 10094. A total of three diets were analyzed, (1) fish fed with *L. lactis* NZ3900 producer of rIFNγ (MT009), (2) fish fed with *L. lactis* NZ3900 containing the plasmid pNZ3900 (MT005), and (3) fish fed with normal feed (NF, and Control). All conditions were challenged with *F. psychrophilum* 10094, with exception of the control. The challenge of fish treated with MT009 and NF was performed in duplicate.

In order to test if the immunostimulation produced by MT009 is able to confer protection against bacterial pathogens, we challenged *O. mykiss* with *F. psychrophilum*. After intraperitoneal injection of *F. psychrophilum* strain 10094, the mortality started at day 6 in all groups challenged with the pathogen and remained similar up to day 10 when survival in the treated group with MT009 stabilized reaching an average of 73.3% at day 17 post-challenge, while the fish treated with MT005 and without treatment reached a 26.6 and 36.6% of survival, respectively. Kaplan–Meier analyses (42) of the survival of fish treated with MT009 showed a statistical difference with the survival curve of fish treated with MT005 (p = 0.0052, Log-rank (Mantel–Cox) Test; p = 0.0169, Gehan–Breslow–Wilcoxon Test) and without treatment (p = 0.0077, Log-rank (Mantel–Cox) Test; p = 0.0180, Gehan–Breslow–Wilcoxon Test). We were unable to identify statistically significant differences in the survival curve of fish treated with MT005 and without treatment. Thus, the Relative Percentage Survival (RPS) of fish treated with MT009 corresponded to 57.8% with respect to fish without treatment (**Figure 6**). The quantification by qPCR of the *F. psychrophilum* (39) in the spleen of survivors and in moribund fish indicated that the former had a bacterial load at least 10 times lower than the moribund fish. The surviving fish without treatment showed a bacterial load that was at least three orders lower than the bacterial load of moribund fish, while surviving fish treated with MT009 showed bacterial loads at least two orders lower than moribund fish (**Supplementary Figure 5**).

### Comparison With Interferon Type II From Salmonids

**Figure 7:**
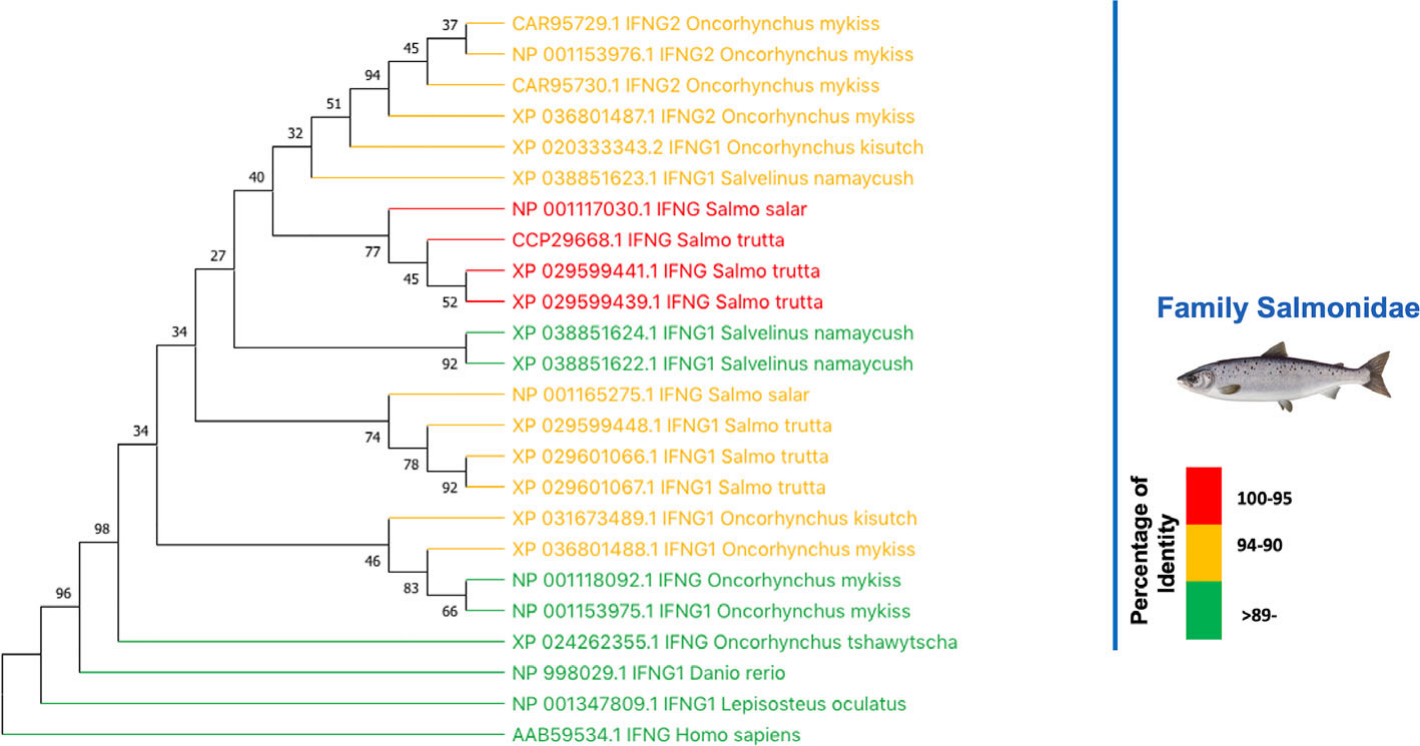
Relationship between type II interferon in salmonids. The figure shows the phylogenetic relationship between the type II interferons of salmonids. The evolutionary history was inferred by using the Maximum Likelihood method and JTT matrix-based model (43). The bootstrap consensus tree inferred from 1,000 replicates (44) is taken to represent the evolutionary history of the taxa analyzed (44). Branches corresponding to partitions reproduced in less than 50% bootstrap replicates are collapsed. The percentage of replicate trees in which the associated taxa clustered together in the bootstrap test (1,000 replicates) is shown next to the branches (44). Initial tree for the heuristic search were obtained automatically by applying Neighbor-Join and BioNJ algorithms to a matrix of pairwise distances estimated using the JTT model, and then by selecting the topology with superior log likelihood value. This analysis involved 24 amino acid sequences. There were a total of 452 positions in the final datasets. Evolutionary analyses were conducted in MEGA X (45, 46). The colors represent the degree of identity of each type II interferon with the IFNγ of *S. salar*.

The results above implies that rIFNγ from *S. salar* can also has biological effect on *O. mykiss*, a salmonid closely related to *S. salar*. Previous studies using IFNγ from *O. mykiss* have indicated that this interferon has a biological effect on *S. salar* kidney cell lines SHK-1, ASK, and TO (17, 21). These findings allow us to hypothesize that the MT009 strain may also have biological effects on other salmonids depending on the degree of relatedness among the type II interferon present in the species. To evaluate this possible range of action, a BLASTP search was performed using the IFNγ sequence of *S. salar* (NP_001117030.1) as query, and a subsequent phylogenetic reconstruction of these results was applied. BLAST analysis indicated that the closest type II interferons were found in *Salmo trutta* with 98.3% (XP_029599439.1) and 97.8% (XP_029599441.1) identity, followed by interferon present in *Salvelinus namaycush* with 94.3% (XP_038851623.1) and *O. mykiss* with 93.3% (CAR95729.1) identity. These results suggest that MT009 could also stimulate the response to type II interferon in *S. trutta* and *S. namaycush* (**Figure 7**).

## Discussion

The current work explores the hypothesis that probiotic microorganisms modified to express type II interferon are able to modify the immune status of the fish and confer protection against pathogens when orally administrated to fish. This protection would result from stimulation of the innate immunity, improving the serological or cellular components. These kind of probiotics, that stimulate the mucosa and beneficially regulate the immune system improving performance against microbial pathogens are termed immunobiotics (47). Some immunobiotics mainly belonging to *Lactobacillus* and *Bifidobacterium* genus, stimulate the expression of interferons (Types I and II) (48, 49) triggering a cascade that confers protection against viral pathogens, such as influenza (50), rotavirus (51) and respiratory syncytial virus (52), and also intracellular parasites like Toxoplasma (53). A common characteristic of these immunobiotics is their capacity to stimulate the cellular immune response mediated by Th1 lymphocytes (48), NK (54, 55) and macrophages (52, 53) which is initiated and amplified by the production of type II interferon.

The type II interferon is a pleotropic cytokine produced mainly by Th1 lymphocytes and NK cells in response to the secretion of IL-12, IL-18, and IL-1β produced by the APC (56). Type II interferon stimulates its own expression on Th1 cells polarizing the Th1/Th2 balance toward a Th1 response (57). Type II interferon also produces a polarization of macrophages M1/M2 balance, favoring the M1 cells phenotype (58). The polarization toward a Th1 and M1 response produces metabolic changes in these cells favoring a glycolytic metabolism, with increased expression of enzymes related to the production of Reactive Oxygen Species (ROS) and Reactive Nitrogen Species (RNS), and the expression of autophagy mediators, especially in M1 macrophages (59–61). These metabolic changes enhance the microbicidal capacity of the cells, improving cell-autonomous immunity against intracellular microorganisms (62). The M1 phenotype of macrophages also secretes IL-6 and TNF-α (63, 64), which in turn induces the expression of serum lysozyme, improving the antimicrobial activity in serum against extracellular pathogens (65, 66).

In teleost, although type II interferon has been much less studied, increasing evidence shows that type II interferon plays a similar role as in mammals sharing activation pathways and functions that are conserved and complexed with paralogous genes of non-redundant function (IFNrel) and diverse receptors (67).

Our work used a Gram-positive bacterial model to express IFNγ, an eukaryotic protein, which is predicted to be glycosylated, as its mammalian counterpart (20). In mammals and teleost specimens like rainbow trout, recombinant IFNγ has been produced in *Escherichia coli* (20). This recombinant IFNγ, has shown to be functional, indicating that glycosylation is not necessary for the activity, although glycosylation increases human interferon half-life protecting against proteolytic degradation (68).

In this work, we detected the rIFNγ mainly in the cytoplasmic fraction of MT009 cultures, despite our original design incorporating an Usp45 signal peptide to allow secretion of the rIFNγ. Low levels of secretion have been also detected in the recombinant production of human (25) and Atlantic salmon type I Interferons (unpublished data) using the Usp45 signal peptide. In human type I IFN this problem was resolved by introducing a spacer sequence between the Usp45 signal peptide and the rest of the interferon protein (25). It remains to determine whether this spacer sequence improves the secretion of rIFNγ by MT009 or not.

Others work have used recombinant overexpression of type II interferon in *E. coli* with further purification to study its function in fish (20). However, *L. lactis*, as an alternative recombinant protein system, may not offer as efficient a protein producer as *E. coli*, and purification of recombinant protein from *L. lactis* cultures may be limited (in yields). To overcome this, in this study we decided to evaluate the ability of different amounts of cytoplasmic extracts of the MT009 strain to stimulate the expression of genes that respond to type II interferon, but taking care to maintain the total amount of bacterial protein constant to evaluate if the effect was caused by the presence of rIFNγ or by some other bacterial component present in the extract.

This approach showed that the genes previously described in rainbow trout and mammalians that respond to type II interferon (Stat1, gamma IP10, IFNγ, and IL-6) (20, 63, 67) are also induced in SHK-1, dose-dependent of the MT009 extract, unlike the gene expression pattern of IL-1β that appeared to respond to the presence of a common component of *L. lactis*.

An interesting point is that *L. lactis* as a Gram-positive bacterium lacks LPS, an inducer of IFNγ expression, that supports that the activation of these genes is due to the presence of rIFNγ in the cytoplasmic extracts of MT009. Although it is not possible to rule out that the overall response is the product of the simultaneous interaction of rIFNγ and some other component of the bacterium. In mammals, type II interferon has been reported to improve the response of keratinocytes and macrophages after stimulation of Toll-Like Receptors (TLR) with microorganisms or agonist, respectively (69, 70).

Once the effect of Type II interferon was tested *in vitro*, we evaluated whether the administration of strain MT009 could induce the expression of genes markers from the response to interferon in the central immune organs of rainbow trout, the spleen and kidney. Although the first encounter of the MT009 strain with the fish immune system is through the digestive mucosa, we hypothesized that the immune cells (lymphocytes and macrophages) present in the GALT respond to type II interferon and stimulate the central immune response, similar to how oral vaccines stimulate the immune response at the local and systemic level (71).

However, the mechanism of how GALT stimulation produces changes at the level of the central immune organs remains poorly understood in teleost. In the case of MT009, this could involve a) stimulation by rIFNγ of the immune cells of the GALT, which in turn responds by secreting cytokines that stimulate the spleen and kidney, b) diffusion of rIFNγ from the intestine to the central immune organs, or both. An interesting point is that during the administration of MT009, we identified an early response in the kidney of the IFNγ response genes, STAT-1, gamma IP10, and IFNγ which was out of phase with the later response observed in spleen. This difference could be the consequence of the presence of a population of immune cells with different activation thresholds by IFNγ or by another cytokine secreted in response to IFNγ. The transient increase in the expression of IFNγ in the spleen and kidney during feeding suggests an initial polarization of lymphocytes to a Th1 phenotype.

After feeding with MT009, changes were also observed in the expression of the genes in response to IFNγ, in particular, an increase in the expression of STAT-1 at 3 days post treatment with MT009. This change in expression could be a consequence of additive effect of rIFNγ and endogenous interferon produced during the administration of MT009. An experiment carried out in our laboratory indicated that *L. lactis* remains in the gut up to 4 days after oral administration has ended (data not shown), a situation that supports the proposal of a combined effect of rIFNγ and endogenous IFNγ.

An interesting point to note is that we also observed a slight increase in IFNγ expression in fish fed with *L. lactis* NZ3900 (MT005), but that it was not associated with a significant increase in other IFNγ response markers. This could be a result of the stimulating effect of *L. lactis*, as occurs in the case of some immunobiotics or lactic acid bacteria in finfish (47, 72). However, in fish fed with MT009, the effect on IFNγ expression was sustained until day 7, which suggests that the effect of rIFNγ administration initiates a cascade that is maintained even after feeding has ended. This is also reflected in the increased expression of IL-6, a cytokine that is secreted by the M1 phenotype of macrophages in mammals and teleosts (73) and also in the increased activity of serum lysozyme, which is induced specifically by strain MT009 and not by MT005.

Taking into consideration that M1 macrophages are producers of IL-6 (73) and lysozyme (65, 66), and that IL-6 also promotes differentiation of monocyte to macrophages in mammals (74), the results suggest that the administration of MT009 promotes macrophages with the phenotype M1 increasing the microbicidal activity at the cellular and humoral level.

This interpretation is consistent with the increase in resistance to extracellular infection such as that produced by *F. psychrophilum*, and potentially could aid the treatment or prevention of infection by intracellular pathogens such as *P. salmonis*. Taking into consideration that the challenge test was carried out by intraperitoneal injection, bypassing the mucosal barriers, the stimulation at the mucosal level by the MT009 strain would improve the ability of the fish to resolve the infection, increasing the fish survival.

The biological functionality of rIFNγ from *S. salar* was tested in *S. salar* cells and in *O. mykiss*, based that the IFNγ of both species presents 93% identity and 96% similarity. It therefore plausible that the MT009 strain could present activity in other salmonids whose type II interferon are closer to that of *S. salar*, such as the IFNγ of *S. trutta*, and *S. namaycush*. It however, remains to be determined whether MT009 actually has a stimulating effect on salmonids other than *O. mykiss* and *S. salar*.

## Conclusions

Our results indicate that in *O. mykiss*, the oral administration of rIFNγ-producing *L. lactis* (MT009) stimulates the expression of genes that participate in the IFNγ-mediated response at the systemic level, producing a state of protection against infection of extracellular bacterial pathogens. This protection could be mediated by polarization towards the Th1 and M1 phenotypes. The process by which stimulation at the mucosal level with MT009 is connected to a change at the systemic level that provides protection remains to be elucidated. Further characterization of the IFNγ response genes and immune cell populations in GALT could help clarify this mechanism.

## Data Availability Statement

The raw data supporting the conclusions of this article will be made available by the authors, without undue reservation.

## Ethics Statement

Ethical review and approval were not required for the animal study because the applied projects financed by CORFO do not require the approval of an ethics committee for their execution.

## Author Contributions

Conceptualization, MT. Methodology, AS, DP, MP, CM, NV, CZ and RV. Investigation, AS, DP, MP, CM, NV, CZ and RV. Resources, MT. Writing—original draft preparation, MT. Writing, review and editing, MT, RV, MP and AG. All authors contributed to the article and approved the submitted version.

## Funding

This research was funded by CORFO-INNOVA, grant number 13CTI21257. The APC was funded by USACH. AS was supported by National Doctorate scholarship no. 21180465 (ANID; Government of Chile).

## Conflict of Interest

Author MT was employed by company Ictio Biotechnologies SA. The authors declare that the research carried out as well as the results present in this work have been in common agreement with Ictio Biotechnologies SA and the licensee Ictiobiotic SpA. Part of these results have been included in the patent application number 2897-2017 requested in the Chilean industrial property registry (INAPI).

The remaining authors declare that the research was conducted in the absence of any commercial or financial relationships that could be construed as a potential conflict of interest.
