# Supplementary material for: Oral Administration of Lactococcus lactis Producing Interferon Type II, Enhances the Immune Response Against Bacterial Pathogens in Rainbow Trout
Source: Front Immunol. 2021 Jun 25;12:696803. doi: 10.3389/fimmu.2021.696803 (PMC8268009; doi:10.3389/fimmu.2021.696803)
Supplement: Supplementary file 1 [file DataSheet_1.docx]

# **Supplementary Material**


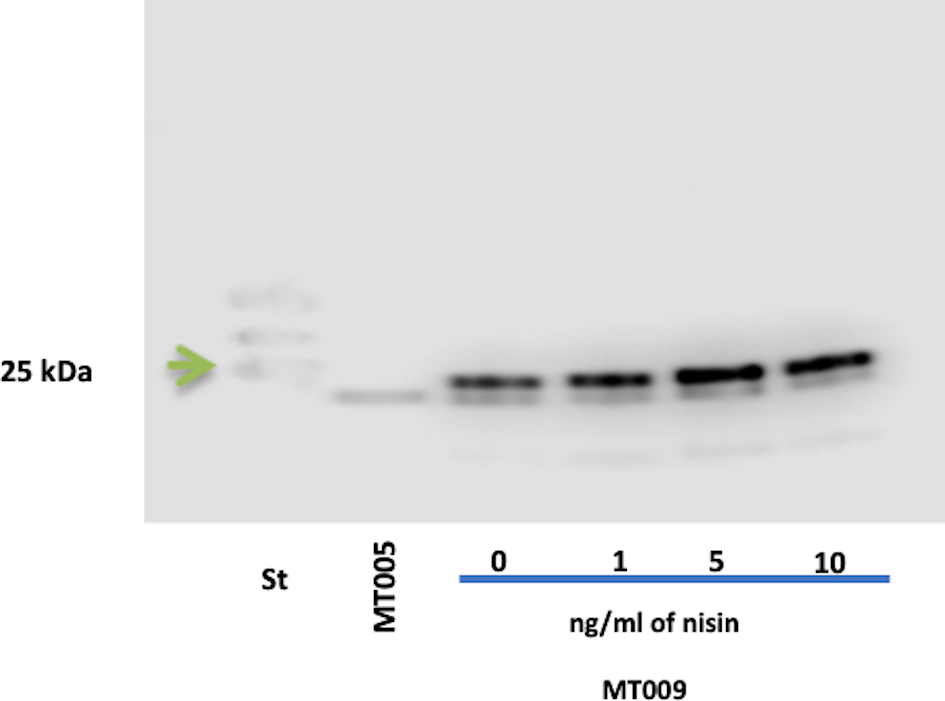


**Supplementary Figure 1**: Effects of nisin concentration on the expression of rIFNγ. The figure shows a western blot using an anti-Histidine tag to analyze the expression of rIFNγ in MT009 (*Lactococcus lactis* NZ3900 prIFNγ) under different concentrations of nisin (0, 1, 5, and 10 ng/ml). A total of 20 μg of protein from the cytoplasmatic extract of MT009 and MT005 (*Lactococcus lactis* NZ3900 with pNZ8149) cultures was loaded in each lane. The green arrow indicates the 25 kDa band of the standard marker (St, left lane)


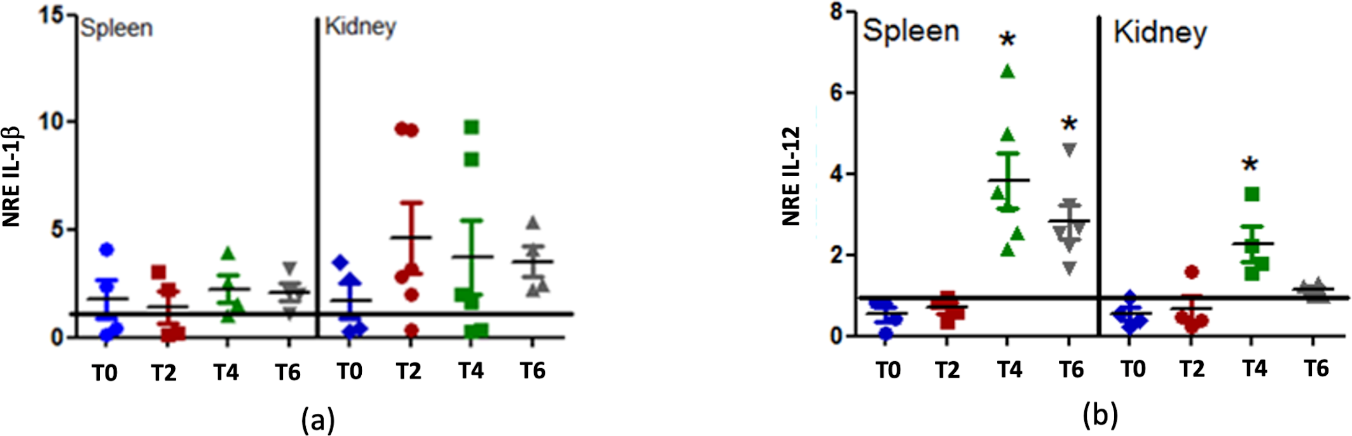


**Supplementary Figure 2**: Expression of IL-1β and IL-12 during the administration of MT009. The figure shows the relative expression of IL-1β (a) and IL-12 (b) in the spleen and kidney at the beginning of the feeding (T0) with MT009 (*Lactococcus lactis* NZ3900 prIFNγ) and at day 2 (T2), day 4 (T4), and day 6 (T6) of MT009 administration. Gene expression was normalized with respect to the expression of the eF1α gene and by the expression of the genes in the control condition. The expression values were compared with the expression at T0. The significance was analyzed using the Mann Whitney test (🞱 p< 0.05).


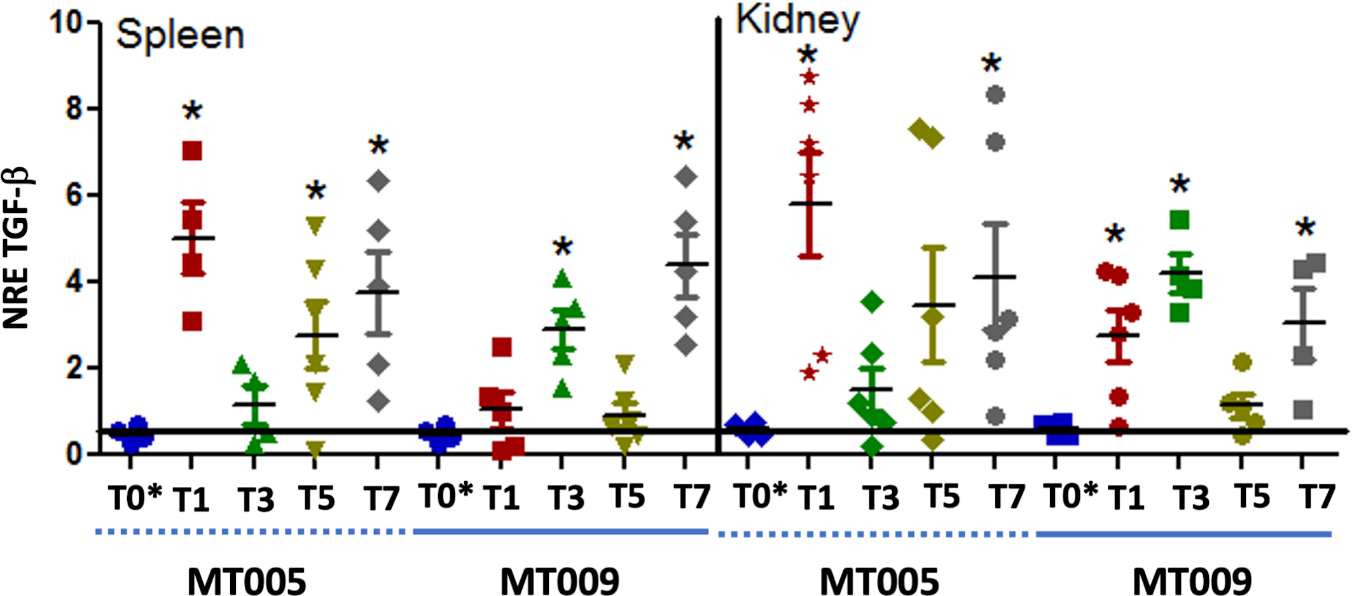


**Supplementary Figure 3**: Expression of TGF-β after administration of MT005 and MT009. The figure shows the relative expression of TGF-β in fish treated with MT009 (*Lactococcus lactis* NZ3900 prIFNγ) and fish treated with the *L. lactis* strain containing the plasmid pNZ8149 (MT005). The expression was evaluated in the spleen and kidney at the beginning of the treatment (*T0) and every two days for 7 days after treatment (T1, T3, T5, T7). The expression was normalized with respect to the expression of the gene encoding for eF1α and by the expression of the genes in the control condition. The expression values along the experiment were compared with the expression in T0. The significance was analyzed using the Mann Whitney test (🞱 p< 0.05).


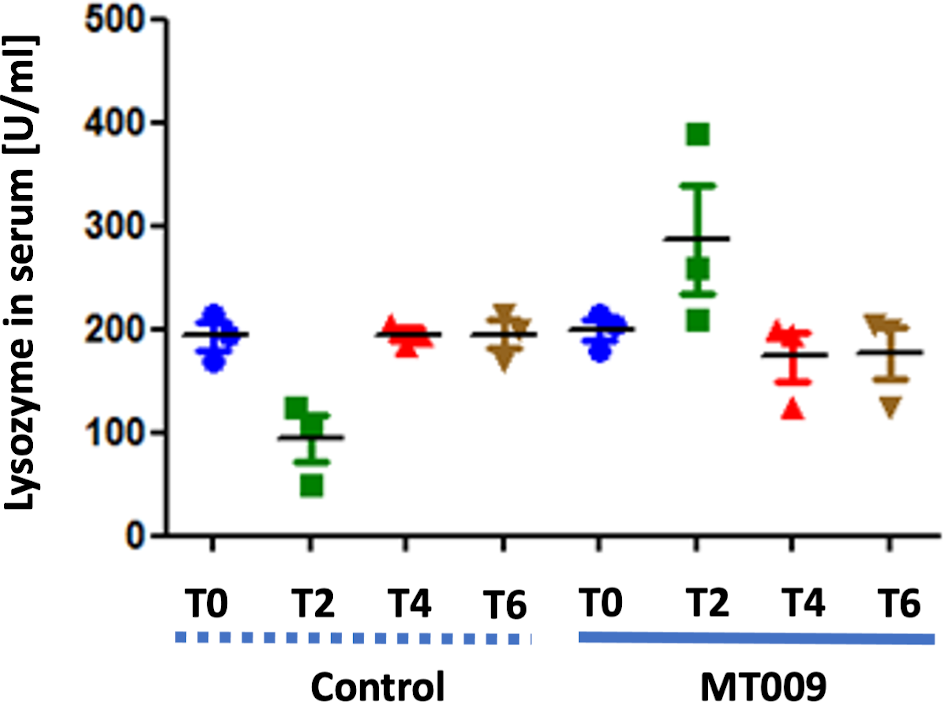


Supplementary Figure 4. Lysozyme activity during administration of MT009 (*Lactococcus lactis* NZ3900 prIFNγ). The figure shows the lysozyme activity in serum from fish sampled at the beginning of the experiment (T0), and at days 2 (T2), 4 (T4), 6 (T6) during the administration of MT009. Control corresponds to fish fed with normal feed.


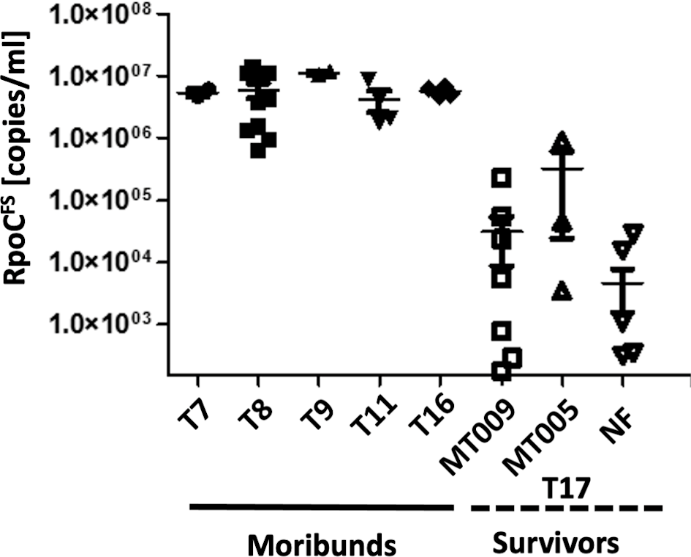


**Supplementary Figure 5**. Bacterial load of *Flavobacterium psychrophilum* in fish tissue. The figure shows the bacterial load of *F. psychrophilum* the spleen of moribund and surviving *Oncorhynchus* mykiss at days 7 (T7), 8 (T8), 9 (T9), 11 (T11), 16 (T16), and 17 (T17) post infection. The bacterial loads of survivors are shown according to the feed applied previous to the challenge including feed supplemented with MT009 (open square, ), MT005 (open triangle, △), or normal feed (NF, open inverted triangle ▽). The bacterial load was calculated from the concentration of *rpoC* copies. The qPCR reaction was performed using 50 ng of total DNA extracted from approximately 30 mg of spleen.
